# Social Daydreaming and Adjustment: An Experience-Sampling Study of Socio-Emotional Adaptation During a Life Transition

**DOI:** 10.3389/fpsyg.2016.00013

**Published:** 2016-01-21

**Authors:** Giulia L. Poerio, Peter Totterdell, Lisa-Marie Emerson, Eleanor Miles

**Affiliations:** ^1^Department of Psychology, University of YorkYork, UK; ^2^Department of Psychology, University of SheffieldSheffield, UK; ^3^School of Psychology, University of SussexBrighton, UK

**Keywords:** daydreaming, mind wandering, socio-emotional adaptation, social cognition, social emotion, emotional inertia, loneliness, experience-sampling

## Abstract

Estimates suggest that up to half of waking life is spent daydreaming; that is, engaged in thought that is independent of, and unrelated to, one’s current task. Emerging research indicates that daydreams are predominately social suggesting that daydreams may serve socio-emotional functions. Here we explore the functional role of social daydreaming for socio-emotional adjustment during an important and stressful life transition (the transition to university) using experience-sampling with 103 participants over 28 days. Over time, social daydreams increased in their positive characteristics and positive emotional outcomes; specifically, participants reported that their daydreams made them feel more socially connected and less lonely, and that the content of their daydreams became less fanciful and involved higher quality relationships. These characteristics then predicted less loneliness at the end of the study, which, in turn was associated with greater social adaptation to university. Feelings of connection resulting from social daydreams were also associated with less emotional inertia in participants who reported being less socially adapted to university. Findings indicate that social daydreaming is functional for promoting socio-emotional adjustment to an important life event. We highlight the need to consider the social content of stimulus-independent cognitions, their characteristics, and patterns of change, to specify how social thoughts enable socio-emotional adaptation.

## Introduction

Throughout life, individuals must adapt to challenges in their environment, which may be minor such as daily hassles, or major such as life transitions and traumatic events (including the transition to university; Shaver et al., 1985). People show remarkable ability to adjust to significant life events (e.g., Frederick and Loewenstein, 1999; Bonanno, 2004) and often do so much faster than they anticipate (Gilbert et al., 1998). How do everyday thoughts help or hinder such adjustment? Cognitive theories of adjustment propose that the content of thinking predicts adaptive or maladaptive outcomes (e.g., Segerstrom et al., 2003; Watkins, 2008). However, these approaches overlook the *social* content of cognition, which is surprising because many events requiring adjustment are social in nature, and the cognitions associated with adjustment are therefore likely to be interpersonally focused. We address this gap and explore how everyday social thoughts in daydreams are related to the process of socio-emotional adjustment in the context of adaptation to a new environment, namely, young adults’ first transition to university.

### Social Daydreaming

When people are not thinking about or processing the external world, their attention turns inward toward self-generated information (e.g., reliving past experiences and formulating future plans). A substantial portion of daily life is occupied with such daydreaming activity; estimates indicate that this may be between 30 and 50% of waking thought (Klinger and Cox, 1987; Killingsworth and Gilbert, 2010). Daydreams represent mental content that is stimulus-independent (i.e., based on memory rather than perception) and task-unrelated (i.e., unrelated to current activity; see Smallwood and Schooler, 2015). Although daydreams are stimulus-independent, their occurrence and content are often influenced by cues in the external environment, such as when certain smells or tastes conjure nostalgic daydreams of childhood. However, the stimulus-independency of daydreaming reflects that fact that, despite sometimes being triggered by stimuli in the external world, the object of attentional focus *during* daydreaming is internal rather than external. Daydreaming is sometimes considered synonymous with spontaneous thought (e.g., Christoff et al., 2004) but we take the view that daydreams can also be deliberate (e.g., Giambra, 1995; Seli et al., 2015) and the extent to which daydreams occur and develop deliberately or spontaneously is likely to be a key dimension upon which daydreams can vary.

Several investigations indicate that daydreams are predominately social in nature (i.e., involving the mental representation of others). A large-scale survey demonstrated that nearly three-quarters of daydreams frequently or always involved others (Mar et al., 2012), other people feature in 71% of daydreaming instances (Song and Wang, 2012), and content analysis of daydreams identifies other-related thoughts as a major thought flow dimension (Andrews-Hanna et al., 2013).

Given the frequency of social daydreaming it would seem likely to serve adaptive socio-emotional functions. Although early texts on daydreaming have speculated on the adaptive value of daydreaming for social relationships and well-being (e.g., Singer, 1966; Klinger, 1990), little empirical evidence exists to support these claims. We suggest that individuals’ social daydreams are central to understanding how individuals adjust to social challenges. During times of social challenge, social daydreams reflect attempts to mentally address pertinent social goals and needs, but whether they do so effectively, and promote later adjustment, depends on the specific characteristics of daydreams (e.g., their emotional content and outcomes, their fanciful nature, and social content) and their dynamics. We review evidence for this proposal showing that (1) environmental challenges bias daydreaming content, (2) daydreaming characteristics predict (mal)adjustment, and (3) social daydreams have beneficial effects on socio-emotional well-being.

### Environmental Challenges Bias Daydreaming Content

When people are faced with challenges in life, particularly emotionally significant ones, their thought flow is dominated by those events. Daydreaming content is biased in this way because daydreams are connected to an individual’s current goals, needs, and desires. This relationship is articulated by Klinger’s (2009, 2013) current concerns theory, which proposes that daydreaming content is dictated by an individual’s current goal pursuits (i.e., desired end states). Daydreams occur when an individual encounters goal-relevant information in situations where overt action toward goals is not possible. Daydreams therefore allow mental progress toward relevant goals, which may involve processing and consolidating current goal pursuits, considering the possibilities and means to achieve them, as well as preparing for and motivating later action (Klinger, 2013).

The proposition that ongoing thought content is dictated by current concerns is supported by empirical studies showing that: daydreaming content and frequency is naturally related to individuals’ important current life concerns and self-relevant goals (Klinger et al., 1980; Gold and Reilly, 1985; Baird et al., 2011; Stawarczyk et al., 2011) and that experimentally manipulating goals or needs biases daydreaming content toward mentally addressing those goals and needs (Antrobus et al., 1966; Kappes et al., 2012b; Stawarczyk et al., 2011).

### Thought Content Predicts (Mal)Adjustment

Cognitive theories of adjustment propose that repetitive thinking predicts adjustment to environmental challenges (e.g., Segerstrom et al., 2003). Various forms of repetitive thought have been identified including worry, rumination, mental simulation, cognitive and emotional processing, and reflection (Watkins, 2008), which have both adaptive and maladaptive outcomes with respect to adjustment and well-being. Post-event cognitive processing (e.g., Bower et al., 1998; Calhoun et al., 2000), emotional processing (e.g., Manne et al., 2007; Hoyt et al., 2013) and reflective thinking (e.g., Burwell and Shirk, 2007; Eisma et al., 2014) predict successful adjustment following stressful events. However, other forms of repetitive thinking, notably rumination and worry, have been associated with negative outcomes in the context of adjustment (e.g., Ehlers et al., 1998; Holeva et al., 2002; Ito et al., 2003; Robinson and Alloy, 2003).

Attempts to integrate the seemingly contradictory effects of repetitive thought have resulted in dimensional approaches, which propose that the positive or negative effects of cognition on adjustment depend on its content (Segerstrom et al., 2003; Watkins, 2008). Several important dimensions have been identified: valence, purpose, and level of construal. Positively valenced repetitive thoughts tend to be associated with positive outcomes, especially when thoughts involve a searching purpose (i.e., exploring possibilities and understanding); negatively valenced repetitive thoughts tend to be associated with negative outcomes, especially when they involve a searching purpose and are abstract (Segerstrom et al., 2003, 2010, 2015; Watkins, 2008)

Similarly, *The Content Regulation Hypothesis* (Smallwood and Andrews-Hanna, 2013) states that the (mal)adaptive impact of daydreaming on psychological well-being depends on its content. For example, daydreaming may be associated with depression only when episodes are unintentional (Deng et al., 2014) or when thoughts are self-focused and indicative of negative rumination (Marchetti et al., 2014). Additionally, daydreams with a positive emotional content (Poerio et al., 2013), future time orientation (Ruby et al., 2013), and content of interest to the daydreamer (Franklin et al., 2013) have all been associated with positive emotional outcomes. Thus, rather than viewing repetitive thoughts or daydreams as inherently adaptive or maladaptive, their effects depend on the content of cognitions and context in which they occur.

Although dimensional approaches have helped to make sense of how thinking can have adaptive and maladaptive outcomes, they do not typically consider the *social* content of thought. As an exception, Segerstrom et al. (2003, Study 2) identified that repetitive thinking can vary to the extent that it is interpersonally or intrapersonally focused and found that the effects of negative repetitive thinking on depression were most pronounced when cognition was self- rather than other-focused (Segerstrom et al., 2003, Study 3). This finding dovetails with the consistent relationship observed between self-focused attention and negative affect (Mor and Winquist, 2002) and indicates that self-focused negative thinking is particularly detrimental. Although self-focused thinking may have negative outcomes, research has yet to fully document the effects of more other-focused thinking on adjustment.

### Social Daydreams and Socio-Emotional Well-Being

Although research has not yet examined how imagining others is related to adjustment, social daydreaming has been linked with positive effects on socio-emotional well-being, in particular, loneliness. Mar et al. (2012) found that although loneliness was associated with more social daydreaming, only the tendency to daydream about close others (versus non-close others) was associated with greater socio-emotional well-being. This suggests that lonely individuals engage in more social daydreaming to counteract loneliness; however, only daydreaming about close others confers a socio-emotional benefit, whereas daydreaming about non-close others may exacerbate loneliness. Likewise, research on imagined interactions—internal dialogs with real-life significant others (Edwards et al., 1988)—suggests the social daydreams of chronically lonely individuals may be indicative of a maladaptive response. Chronically lonely individuals report experiencing fewer, less satisfying, and more negative imagined interactions (Honeycutt et al., 1989) suggesting that loneliness may be exacerbated by a lack of positive social daydreaming and, by extension, that frequent and positive social daydreams may buffer against loneliness.

Studies examining the effects of individual social daydreams on momentary socio-emotional well-being also show that certain types of social daydreaming can regulate and shape emotional well-being. Poerio et al. (2015b) found that social, but not non-social, daydreams were associated with increased feelings of happiness, love, and connection with others. Interestingly, this association was only observed when participants were low in these feelings before daydreaming and when their daydreams involved close others implying that daydreams about close others may function to regulate social emotions. Consistent with this, directed daydreaming about close others has been shown to promote positive social feelings (connection, love, belonging) after induced loneliness (Poerio et al., 2015a) showing that social daydreaming can replenish feelings of interpersonal connection under conditions where belongingness has been threatened.

### Can Social Daydreams Naturally Enhance Socio-Emotional Adjustment?

Although previous research is consistent with the proposal that positive characteristics of social daydreaming may promote socio-emotional adjustment, they are limited, because they do not examine social daydreaming during the process of adjustment. Cross-sectional research has examined the daydreams of individuals, who are currently adapted or maladapted (e.g., lonely or not) and measured supposedly stable and global daydreaming features. This assumes that individuals display consistent patterns of daydreaming over time, does not account for the dynamic nature of daydreaming, and cannot capture the *process* of adaptation over time. Adaptation is often examined as a state rather than a process (Luhmann et al., 2012) because cross-sectional approaches typically examine predictors of adjustment (e.g., repetitive thinking) and levels of adjustment on a particular variable of interest (e.g., depression, loneliness) at the same single point in time. The problem with this approach is that associations (e.g., between daydreaming and loneliness) may be bi-directional or amenable to third-variable explanations, which highlights the need for prospective studies that examine daydreaming repeatedly over time. Although experimental designs have enabled consideration of daydreaming within a specific context (e.g., induced loneliness), daydreaming is artificially directed rather than spontaneously occurring and therefore does not capture the idiosyncratic and personally meaningful nature of daydreaming. Laboratory experiments are also only able to examine the short-term effects of daydreaming, which, again, cannot capture the *process* of adjustment over time. Adjustment is a temporal process, which means that to properly understand, how social daydreams are related to adjustment, it is necessary to capture repeated observations of daydreaming over time in a situation where adjustment is required.

In addition to examining daydreaming as a dynamic process it is also necessary to consider daydreaming as heterogeneous and measure the characteristics of daydreams. The previously reviewed literature suggests some important social daydreaming characteristics that might be expected to predict adjustment: their emotional outcomes (connection, loneliness, and positivity), valence, and the relationship quality between the daydreamer and the most central other person involved in the daydream. Other literature also suggests that the fanciful nature of daydreams may relate to adjustment because fanciful thinking has been previously associated with negative outcomes (e.g., Oettingen and Wadden, 1991; Kappes et al., 2012a).

Consistent with dimensional approaches, social daydreams *per se* should not predict adjustment, but their characteristics and patterns of change over time should. If social daydreams were part of an adaptive response then they should, over time, become more constructive. Specifically, they should be associated with more positive emotional outcomes (greater connection and positivity, and less loneliness), become more positively valenced, involve higher quality relationships, and become less fanciful. This pattern of constructive change over time should then predict later socio-emotional adjustment.

### The Present Study

To capture the dynamic and heterogeneous nature of social daydreams over time and their relationship to adjustment, we used experience-sampling methodology to sample social daydreams during a period of naturally occurring adjustment. Life events offer opportunities to study adjustment, because they are episodes that involve a substantial change in an individual’s daily routine and require a new behavioral response. We chose to examine social daydreaming during students’ first transition to university because it is (1) a stressful life event that requires an adaptive response; it is reported as one of the most stressful periods of adjustment in life (Shaver et al., 1985) and is associated with increased psychological ill-health (e.g., Bewick et al., 2010) and (2) a time of *socio-emotional* challenge where social goals and emotions (e.g., preventing loneliness) are likely to be important, perhaps more so than academic or financial ones (Arthur and Hiebert, 1996; Bitsika et al., 2010), because moving to university disrupts existing social support networks and requires the formation of new relationships.

To examine, how social daydreaming was related to socio-emotional adjustment during this transition, we measured the characteristics of social daydreams described above (i.e., emotional outcomes, valence, relationship quality, fanciful nature) twice daily for 1 month. We measured adjustment outcomes (loneliness and social adjustment to university) after 2 and 4 weeks of the study. We chose these measures to index adjustment at two time points in the study (although other outcomes indicative of adjustment such as subjective well-being or levels of depression might also have been warranted) due their relevance to the particular transition and the socio-emotional aspects of that transition, which was our main focus. The social adaptation to university scale measures the extent to which students feel that they have integrated socially to university life and is therefore directly relevant for capturing perceptions of social adjustment within the study context. The measurement of loneliness is an adjustment outcome consistent with previous daydreaming research and pertinent to the university transition because it has been identified as a commonly experienced feeling and issue for transitioning students, particularly in the first months of the transition (Cutrona, 1982; Shaver et al., 1985). The repeated measurement of social daydreaming and its characteristics enabled an examination of the temporal process of daydreaming during adjustment to university.

#### Daydreaming Over Time

We predicted that positive features of daydreaming would increase over time indicative of adjustment. However, we reasoned that because the process of adjustment is likely to first involve participants’ reaction to the new environment followed by an adaptive response, evidence of positive change over time for social daydreams would be delayed. For this reason, we examined how social daydreams changed over time during the earlier and later stages of the transition separately. We expected social daydreams to show positive and constructive patterns of change in the last weeks (when students are adapting) but not the first weeks (when students are reacting). We also examined how feelings in general changed over time, specifically feelings of connection, positivity, and negativity. Like social daydreaming, we expected feelings to change over time, becoming increasingly positive, but only in the last weeks of the study.

#### Emotional Inertia as an Index of Adjustment

As additional evidence of the role of social daydreams in adjustment, we drew on the concept of emotional inertia. Emotional inertia describes resistance to emotional change over time and is thought to reflect psychological maladjustment (Kuppens et al., 2010). Emotional inertia can be indexed by the extent to which a person’s current emotional state is predicted by their emotional state at a previous time point (i.e., the autocorrelation between successive measurements of emotional states). Evidence that high emotional inertia is indicative of a maladaptive response comes from studies demonstrating that emotional inertia predicts depression (Kuppens et al., 2012) and ill health (Wang et al., 2012). We reasoned that if social daydreams were linked to successful adjustment, then we would expect the emotional outcomes of daydreaming to show less evidence of inertia (i.e., show faster changes in the emotional outcomes of daydreams) in individuals who are currently socially maladjusted to the university transition (i.e., participants who report being less socially integrated within university than others).

#### Social Daydreaming and Later Adjustment

Based on daydreaming research, we also predicted that positive features of social daydreaming would predict better socio-emotional adjustment. We used loneliness and social adaptation to university to index adjustment, and measured them after 2 (T1) and 4 (T2) weeks. If, as we propose, social daydreams promote socio-emotional adjustment, then the positive features of social daydreams should predict better adjustment at T2 controlling for T1.

## Materials and Methods

### Participants

One hundred and three first year students at a UK university (*M*_age_ = 19.34, *SD* = 2.34; 75 females) were recruited to the study, which was described as an investigation into first year undergraduates’ thoughts and feelings. Sample size was based on recommendations that at least 100 groups at level-2 (i.e., participants in the current study) should be used when analyzing data with multi-level structural equation modeling (Hox et al., 2010). Students were recruited at the start of their first year of university via email advertisement, flyers, word of mouth, and participant referrals. All first year undergraduate students could take part on the condition that they had access to a smartphone with Internet access for the duration of the study. Participants started the study during the first 3 weeks of the first semester of university (September 22nd, 2014 – October 13th, 2014), which comprised of introduction week and the first two teaching weeks of the semester. In exchange for participation, psychology undergraduates (59%) were given course credits; non-psychology students were given £10 worth of food vouchers. Ethical approval for the study was obtained from the University of Sheffield Psychology ethics committee and informed consent was obtained from all participants before the study began.

### Procedure

Participants attended a group training session where they were given written and verbal instructions for the study. Daydreaming was defined as stimulus-independent and task-unrelated thought. Social/non-social daydreams were defined as daydreams where another (real or imaginary) person or people are/are not involved. Participants were given a demonstration of the experience-sampling method, a verbal explanation of the meaning and response to each questionnaire item and instructions on how to complete the survey.

**FIGURE 1:**
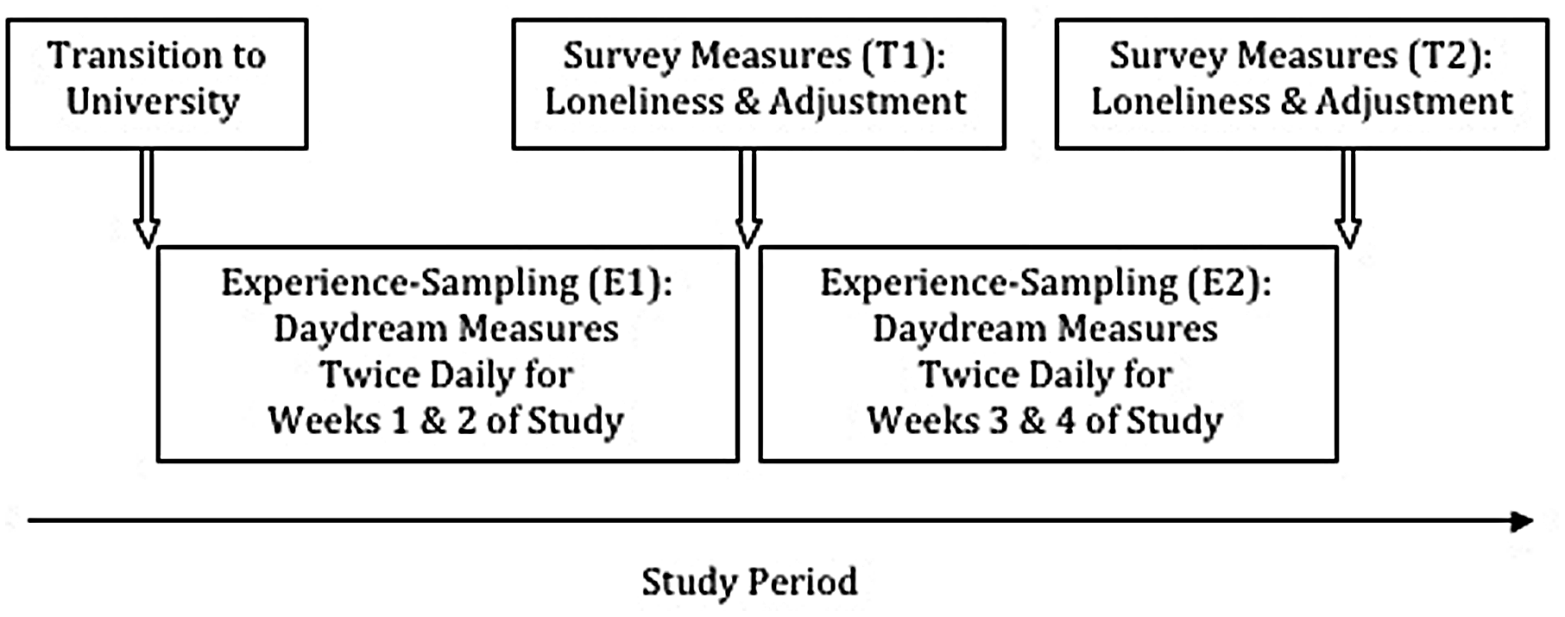
**Study protocol**.

**Figure 1** summarizes the study design timeline. The first experience-sampling period (E1) began the day after the training session and the second experience-sampling period (E2) began 2 weeks later. Overall, participants were signaled twice daily over 28 days via text message to their smartphones and reported on their current or most recent social daydream by answering an online questionnaire using their smartphone. Participants received the signals at random times each day between 10:00 and 22:00 (one between 10:00 and 16:00, the other between 16:00 and 22:00 with at least 1 h between consecutive signals). Randomization of signals was used to prevent anticipation and to sample daydreams across a range of times and daily activities. Previous experience-sampling approaches in the daydreaming literature have signaled participants more frequently but for a shorter period of time (e.g., 8 and 12 signals daily for 7 days, Kane et al., 2007; Poerio et al., 2013). Decisions about sampling protocols in experience-sampling designs typically reflect a balance between the frequency of signals, study duration, and time to complete measures in order to minimize participant burden (Christensen et al., 2003). Given our interest in change over a relatively long period of time (28 days) we employed a low sampling frequency rate (twice daily) so as not to place excessive demands on participants. Participants also completed two online questionnaires, prompted via email, at the end of the first 2 weeks of the study (T1) and at the end of the study (T2), which measured loneliness and social adaptation to university over the past 2 weeks.

### Experience-Sampling Measures

#### Daydreaming

Participants answered: “*Right before you were signaled, were you daydreaming?*” (“*Yes*” = 1, “*No*” = 0). If they answered affirmatively, then they answered: *“Did your daydream involve another person or people?”* (“*Yes*” = 1, “*No*” = 0). When participants did not experience a social daydream immediately prior to signaling, they were instructed, *“Please think about your last daydream that involved another person or people.”* Participants then answered the questions described below in a randomized order.

#### Daydreaming Characteristics

Participants rated how their daydream made them feel compared to before it for three measures of emotion using 7-point response scales (1 = *much less*, 4 = *no different*, 7 = *much more*). Single items measured social connection (“*connected*”), social disconnection (“*lonely*”) and a single item measured positive feelings (“*positive*”) in response to the daydream. Participants rated the valence of their daydream from 1(*very negative*) to 7(*very positive*) and how fanciful their daydream was from 1(*completely realistic*) to 7(*completely fanciful*). Participants also rated the quality of the relationship between themselves and the most central other person involved in their daydream. Three items, based on previous research (Niven et al., 2012), were used to index relationship quality: participants rated their general feelings of closeness, liking, and trust toward that person on scales from 1(*not at all*) to 7(*extremely*). Items were averaged to create an overall score for relationship quality (α = 0.90).

#### Feelings from Environment

Participants rated the extent to which they had felt “*connected with others*,” “*positive*,” and “*negative*” so far that day, or since their last signal on that day on scales from 1(*not at all*) to 7(*a great deal*).

### T1 and T2 Measures

#### Loneliness

Loneliness was measured using the eight-item short-form of the UCLA Loneliness Scale (ULS-8; Hays and DiMatteo, 1987). Participants rated the extent to which they had felt socially isolated over the past 2 weeks (e.g., “*Isolated from others*”) from 1(*never*) to 4(*always*). Items were averaged to provide a score for loneliness, with higher scores indicating greater loneliness. Internal reliability was high at T1 (α = 0.89) and T2 (α = 0.91).

#### Social Adaptation to University

This was measured using the 20-item social adjustment subscale of the Student Adjustment to College questionnaire (SACQ; Baker and Siryk, 1989). Participants were asked to consider the “past 2 weeks” when indicating the extent to which several items indicating social adaptation (e.g., *“I am very involved in social activities in university”)* apply to them from 1 (*applies very closely to me*) to 9 (*doesn’t apply to me at all*). Negatively worded items were reverse-scored and items were then averaged to create an overall score for social adaptation to university with higher scores indicating greater social adjustment. Internal reliability was high at T1 (α = 0.93) and T2 (α = 0.93).

## Results

### Response Rate

Participants completed 3697 out of a possible 5768 responses corresponding to a 64% response rate. On these occasions, participants reported that they were currently daydreaming 64% of the time and 92% of these daydreams were social. Of the social daydreams during the study, 46% involved people before university, 48% involved people after university, and 6% involved some other category (e.g., an imaginary person, celebrity). Social daydreams showed a slight shift to after-university contacts during the later weeks: during E1, 47% of social daydreams involved people before university, 45% involved people after university, and 8% involved some other category; during E2, 45% of social involved people before university, 50% involved people after university, and 5% involved some other category. The percentage of social daydreams in the present study is notably higher than other estimates (e.g., 71%; Song and Wang, 2012). This fits well with the proposal that social daydreams become more frequent during times of social challenge. When participants were not daydreaming at the time of signaling, or if their current daydream was not social, then they reported on their last social daydream, which occurred on 1532 occasions (41%). Current or last social daydreams (coded 0, 1) did not show different associations to any of the experience-sampling dependent variables (i.e., the emotional content and outcomes of daydreaming, their fanciful nature, the relationship quality, and feelings from the environment did not differ according to whether daydreams were currently experienced or reported retrospectively). We interpret this as evidence that retrospective and in the moment reports of social daydreams were reported with similar levels of accuracy by participants.

Ninety-nine participants completed the T1 questionnaire (a 96% response rate); at this stage two participants had dropped out of the study because they had left university, and one participant could not continue owing to difficulty tracking daydreaming experience. Ninety-seven of the 99 participants who completed the T1 questionnaire also completed the T2 questionnaire (a total response rate of 94%).

### Did the Emotional Outcomes and Content of Daydreams and Feelings from the Environment Change Over Time?

To examine whether daydreams and feelings in general showed significant patterns of change over time, we examined the effect of time on each dependent variable from the experience-sampling measures. The data had a natural two-level structure (i.e., responses collected over a series of time-points nested within individuals) so data were analyzed by multi-level modeling (Hox, 2010) using the Mixed procedure in IBM SPSS v.21 software. We examined the effect of time on daydreaming and feelings separately for the first and second experience-sampling periods (E1 and E2). The within and between subjects variance of each dependent variable was partitioned by fitting random intercept and slope terms for each individual. Non-independence of observations was modeled by fitting an autoregressive correlation structure (AR1) to level-1 residuals. Time since starting the study was tested as a fixed effect. Some participants began the study later than others so we created a variable representing lapsed time since starting university on commencing the study and entered this as a fixed effect in all models to control for its potential influence. Time since starting the study was non-significant in all models except for predicting how lonely participants’ social daydreams made them feel during E1. Specifically, participants’ who started the study later, had daydreams that made them feel less lonely during E1 [*B* = –0.02, *t*(101) = –2.09, *p* = 0.039].

**Table 1** summarizes the effect of time on social daydreaming and feelings from the environment for E1 and E2. The first weeks of the study (E1) were not characterized by any significant patterns of change for the characteristics of daydreaming. For more general feelings from the environment, feelings of connection with others and feeling positive did not show significant patterns of change. However, feeling negative showed a significant and reliable increase during E1 [β = 0.06, *B* = 0.00836, *t*(428) = 2.33, *p* = 0.020, 95% CI[0.00132, 0.0154]], suggesting that the first weeks of university may have been a time associated with increased negative emotion.

**Table 1:** Fixed effects of time on daydreaming characteristics and feelings in general over E1 and E2.

	E1						E2					
		
Fixed effects	*df*	β	*B*	*SE*	*t*	*ICC*	*df*	β	*B*	*SE*	*t*	*ICC*
**Emotional outcomes of daydreaming**												
Connected	618	0.00	–0.000327	0.00441	–0.07	0.17	575	0.07	0.0125	0.00388	3.22^∗∗∗^	0.28
Lonely	585	0.01	0.00253	0.00471	0.54	0.22	573	–0.07	–0.0123	0.00412	–2.98^∗∗^	0.30
Positive	592	0.02	0.00483	0.00497	0.97	0.18	571	0.03	0.00592	0.00431	1.37	0.23
**Daydreaming content**												
Valence	596	0.03	0.00756	0.00548	1.38	0.15	601	0.04	0.00711	0.0260	1.45	0.17
Fanciful	612	0.03	0.00850	0.00608	1.40	0.23	534	–0.06	–0.0154	0.00593	–2.60^∗∗^	0.28
Relationship quality	615	0.00	–0.000375	0.00553	–0.07	0.18	607	0.08	0.0168	0.00457	3.69^∗∗∗^	0.25
**Feelings from environment**												
Connectedness	546	–0.01	–0.00166	0.00333	–0.49	0.24	489	0.06	0.00777	0.00325	2.40^∗^	0.35
Positive	542	–0.02	–0.00330	0.00335	–0.99	0.24	442	0.07	0.00898	0.00335	2.68^∗∗^	0.33
Negative	528	0.06	0.00836	0.00358	2.33^∗^	0.31	475	–0.05	–0.00736	0.00330	–2.23^∗^	0.39


*SE, standard error. Time since starting university entered as a fixed effect in all models. ^∗^*p* < 0.05, ^∗∗^*p* < 0.01, ^∗∗∗^*p* < 0.001.*

As expected, the pattern of change over time was substantially different for E2. Over time, participants’ social daydreams made them feel significantly more connected [β = 0.07, *B* = 0.0125, *t*(575) = 3.22, *p* = 0.001, 95% CI[0.00484, 0.0201]] and less lonely [β = –0.07, *B* = –0.0123, *t*(573) = –2.98, *p* = 0.003, 95% CI[–0.0204, –0.00418]], but not more positive [β = 0.03, *B* = 0.00592, *t*(571) = 1.37, *p* = 0.170, 95% CI[0.00255, –0.0144]]. Participants’ social daydreams also became significantly less fanciful in content [β = –0.06, *B* = –0.0154, *t*(534) = –2.60, *p* = 0.009, 95% CI[–0.0271, –0.00379]] and involved higher quality relationships [β = 0.08, *B* = 0.0168, *t*(607) = 3.69, *p* < 0.001, 95% CI[0.00787, 0.0258]]; but did not become more positively valenced [β = 0.04, *B* = 0.00711, *t*(601) = 1.45, *p* = 0.146, 95% CI[–0.00249, 0.0167]]. Likewise, participants reported feeling in general significantly more connected with others [β = 0.06, *B* = 0.00777, *t*(489) = 2.40, *p* = 0.017, 95% CI[0.00140, –0.0142]], more positive [β = 0.07, *B* = 0.00898, *t*(442) = 2.68, *p* = 0.008, 95% CI[0.00240, 0.0156]] and less negative [β = –0.05, *B* = –0.00736, *t*(475) = –2.23, *p* = 0.026, 95% CI[–0.0138, –0.000867]] over time. We repeated these analyses for two subgroups: early study starters (<2 weeks of starting university, *n* = 55) and late starters (>2 weeks of starting university, *n* = 48). In line with the idea that the adaptive response takes time, effects were most evident for the late starter group during E2. We also observed additional effects for valence such that daydreams for late starters during E2 became more positive over time.

We also repeated these analyses considering the whole sampling period (i.e., 4 weeks). Consistent with the results for E2, over the whole study period, participants social daydreams made them feeling increasingly connected [β = 0.08, *B* = 0.00771, *t*(63) = 3.38, *p* < 0.001, 95% CI[0.00477, 0.0107]], less lonely [β = –0.06, *B* = –0.00635, *t*(69) = –3.00, *p* = 0.004, 95% CI[–0.00954, –0.00317]], and involved higher quality relationships [β = 0.07, *B* = 0.00822, *t*(63) = 3.41, *p* = 0.001, 95% CI[0.00464, 0.0118]]. In contrast to the results from E2 participants’ social daydreams made them feel increasingly positive [β = 0.07, *B* = 0.00718, *t*(64) = 3.58, *p* = 0.001, 95% CI[0.00389, 0.0105]], the content of social daydreams became more positively valenced [β = 0.07, *B* = 0.00704, *t*(60) = 2.61, *p* = 0.012, 95% CI[0.00335, 0.0107]], but did not become less fanciful [β = 0.02, *B* = 0.00320, *t*(72) = 1.63, *p* = 0.107, 95% CI[–0.00108, 0.00747]]. In general, participants also reported feeling increasingly positive [β = 0.04, *B* = 0.00254, *t*(63) = 2.09, *p* = 0.037, 95% CI[0.000186, 0.00488]] and less negative [β = –0.04, *B* = –0.00324, *t*(63) = –2.50, *p* = 0.015, 95% CI[–0.00569, –0.000788]] but not more connected with others [β = 0.04, *B* = 0.00264, *t*(52) = 0.80, *p* = 0.426, 95% CI[–0.000234, 0.00505]] over the study period. Thus the results of the effect of time on social daydreaming and feelings also emerged over the study period, although examinations of E1 and E2 separately indicate that these effects occur, as predicted, later during the transition.

### Emotional Inertia

We predicted that participants, who reported being currently less adjusted to university would show faster changes in the emotional outcomes of their social daydreams (i.e., low emotional inertia) than those who were more adjusted. Given that the social emotional outcomes (connected, lonely) of participants’ daydreams increased significantly during E2, we were interested in examining the extent to which they might show resistance or susceptibility to change depending on levels of social adaptation at T1. Evidence for this would be provided by a significant cross-level interaction between the autocorrelation of each dependent variable (i.e., the lag of the variables for connected and lonely) and levels of social adaptation (results are summarized in **Table 2**).

**Table 2:** Emotional inertia analyses for socio-emotional outcomes of social daydreaming during E2 with social adaptation at T1.

Emotional outcome	Key variable	–2^∗^LL	–2^∗^LLΔ	*df*	Estimate (*SE*)	*p*	95% CI	*ICC*
**Connected**								
Fixed effects	Lag of connected	5357.48	—	1760	0.04 (0.02)	0.075	–0.00, 0.09	0.26
Random effects	Lag of connected	5337.22	20.25^∗∗∗^	86	0.04 (0.01)	0.005	0.02, 0.07	0.03
Level-2 fixed effect	Social adjustment	5314.46	22.76^∗∗∗^	64	0.22 (0.06)	0.001	0.09, 0.34	0.48
Interaction	Lag of connected^∗^Social adaptation	5312.76	1.70	64	0.08 (0.03)	0.008	0.02, 0.14	0.44
**Lonely**								
Fixed effects	Lag of lonely	5540.30	—	1758	0.05 (0.02)	0.021	0.01, 0.10	0.27
Random effects	Lag of lonely	5540.17	0.14	53	0.00 (0.01)	0.764	0.00, 1.44	0.00


*SE, standard error; LL, log likelihood. ^∗∗∗^*p* < 0.001.*

**FIGURE 2:**
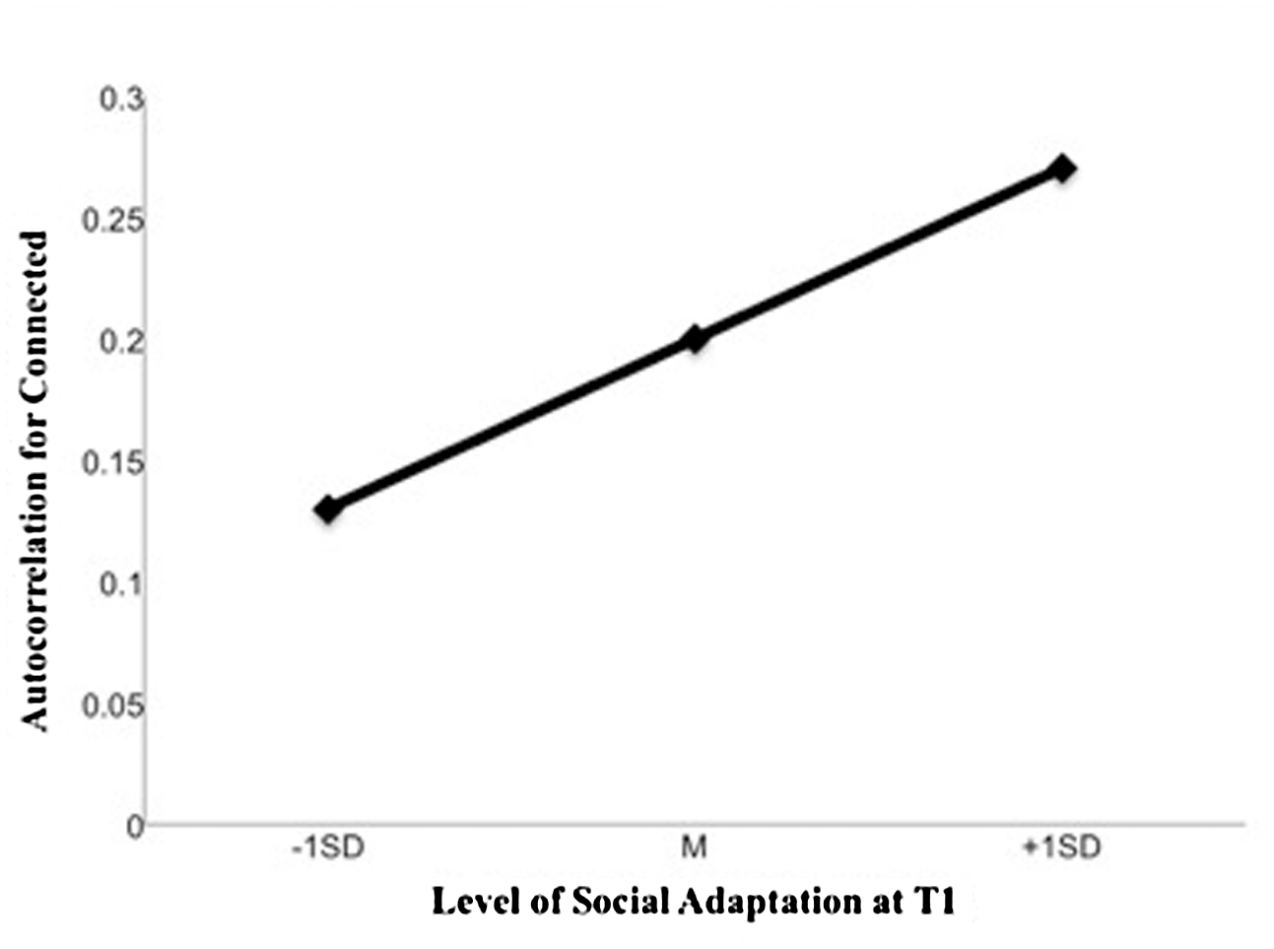
**Emotional inertia for how connected social daydreams made participants’ feel during E2 according to T1 levels of social adaptation**.

The fixed effects of the lag variables on feeling connected and lonely as an index of inertia were positive and significant demonstrating autocorrelation between adjacent time points. The addition of our level-2 predictor was justified by examining the improvement in model fit by allowing slopes as well as intercepts to vary. Improvement in model fit was only significant for the model predicting feeling connected but not lonely. We therefore only examined the cross-level interaction between social adaptation and the lag of connectedness which, as expected, was significant [β = 0.08, *t*(64) = 2.72, *p* = 0.008, 95% CI[0.02, 0.14]]. Equivalent results were obtained when the lag of connectedness was cluster mean centered (β = 0.07, *p* = 0.024). See Hamaker and Grasman (2015) for a discussion of centering in inertia analyses. As shown in **Figure 2**, participants who were low (1SD below the mean), compared to high (1SD above the mean), in social adaptation showed lower levels of inertia. This suggests that participants who were less socially adapted to university showed less inertia for feelings of connection as a result of their social daydreams, indicative of an adaptive response.

### Did Social Daydreaming Predict Loneliness and Social Adaptation to University?

Given the significant patterns of change observed in social daydreaming during E2, we examined whether these daydreaming characteristics predicted later loneliness and social adaptation to university. These analyses required an examination of bottom-up effects (i.e., predicting level-2 outcomes from level-1). Traditional multi-level models do not allow level-2 variables as outcomes (only as predictors), which would not allow us to test, for example, whether loneliness at the end of the study (a level-2 variable) is predicted by daydreaming characteristics during the study (level-1 variables). To overcome this, we therefore used multi-level structural equation modeling (MSEM; Preacher et al., 2010) using Mplus software (Muthén et al., 1998–2011), which can accommodate level-2 variables as outcome variables and mediators. Mplus does not currently allow the modeling of autocorrelation by fitting an autoregressive correlation structure (Bolger and Laurenceau, 2013) so we entered the lag for each level-1 variable within the models (e.g., Totterdell et al., 2006).

#### Loneliness

We examined the effect of daydreaming during E2 on T2 loneliness, controlling for T1 loneliness in all models (T1 loneliness significantly predicted T2 loneliness; all βs > 0.81, all *p*s < 0.001). Results showed that T2 loneliness was negatively predicted by daydreams that made participants feel connected (β = –0.16, *SE* = 0.07, *p* = 0.020, 95% CI[–0.30, –0.05]) and positive (β = –0.20, *SE* = 0.08, *p* = 0.010, 95% CI[–0.33, –0.07]), and positively predicted by daydreams that made participants feel lonely (β = 0.13, *SE* = 0.07, *p* = 0.041, 95% CI[0.03, 0.24]). Likewise, T2 loneliness was negatively predicted by daydreams that were positively valenced (β = –0.24, *SE* = 0.07, *p* = 0.001, 95% CI[–0.36, –0.13]) and involved high quality relationships (β = –0.12, *SE* = 0.06, *p* = 0.042, 95% CI[–0.21, –0.02]), but was positively predicted by fanciful daydreams (β = 0.12, *SE* = 0.05, *p* = 0.021, 95% CI[0.03, 0.21]). This indicates that participants were less lonely at T2 if their daydreams during E2 made them feel more connected, less lonely, and more positive, and their daydreams were less fanciful, more positively valenced and involved higher quality relationships.

#### Social Adaptation

Using the same analytical procedure, we examined the effect of daydreams during E2 on T2 social adaptation to university controlling for T1 social adaptation (T1 social adaptation significantly predicted T2 social adaptation in all models, βs* >* 0.25, all *p*s < 0.05, except when examining relationship quality where this relationship was marginal, β = 0.27, *SE* = 0.14, *p* = 0.062). Unexpectedly, the social daydreams during E2 did not predict T2 social adaptation: social adaptation was not significantly predicted by the emotional outcomes of social daydreams (connected: β = 0.15, *SE* = 0.32, *p* = 0.642, 95% CI[–0.38, 0.67], lonely: β = 0.35, *SE* = 0.33, *p* = 0.292, 95% CI[–0.19, 0.89], positive: β = –0.12, *SE* = 0.30, *p* = 0.687, 95% CI[–0.60, 0.37]) or their characteristics (valence: β = 0.27, *SE* = 0.27, *p* = 0.312, 95% CI[–0.17, 0.71], fanciful: β = 0.07, *SE* = 0.22, *p* = 0.738, 95% CI[–0.43, 0.29], relationship quality: β = 0.03, *SE* = 0.38, *p* = 0.929, 95% CI[–0.59, 0.67]).

#### Supplementary Mediation Analysis

Given that social daydreams were significantly related to loneliness but had no direct effect on social adaptation, we wondered whether social daydreaming might indirectly influence social adaptation through its demonstrated effects on loneliness. To examine this, we constructed a series of multi-level mediation models to examine whether social daydreams during E2 had indirect effects on social adaptation via loneliness. In each model, we controlled for T1 loneliness and T1 social adaptation to university and included the lag of each associated level-1 variable. The results of our multi-level mediation analyses are summarized in **Table 3**.

**Table 3:** Summary of multi-level mediation models examining the indirect effect of social daydreaming characteristics on social adaptation to university via loneliness.

	Path *a*				Path *c*				Path *ab*			
			
	β	*SE*	*p*	95% CI	β	*SE*	*p*	95% CI	β	*SE*	*p*	95% CI
**Emotional outcomes of daydreaming**												
Connected	–0.37	0.18	0.044	–0.67, –0.07	0.01	0.13	0.922	–0.21, 0.23	0.12	0.06	0.047	0.02, 0.22
Lonely	0.39	0.21	0.063	0.05, 0.73	–0.04	0.14	0.799	–0.27, 0.20	–0.13	0.07	0.077	–0.24, –0.01
Positive	–0.42	0.19	0.026	–0.73, –0.11	0.00	0.12	0.991	–0.19, 0.20	0.14	0.06	0.033	0.03, 0.24
**Daydreaming content**												
Valence	–0.50	0.19	0.007	–0.81, –0.19	–0.12	0.12	0.398	–0.31, 0.10	0.16	0.07	0.013	0.06, 0.27
Fanciful	0.60	0.25	0.017	0.19, 1.01	0.34	0.19	0.070	0.03, 0.66	–0.19	0.09	0.024	–0.34, –0.05
Relationship quality	–0.31	0.27	0.244	–0.76, 0.13	–0.12	0.17	0.500	–0.40, 0.17	0.10	0.09	0.250	–0.04, 0.25


*SE, standard error. All models include loneliness and social adaptation at T1 and the lag of the associated level-1 variable. Path *a* represents the effect of daydreaming on loneliness at T2, path *c* represents the effect of daydreaming on social adaptation at T2, and path *ab* represents the indirect (mediated) effect of daydreaming on social adaptation via loneliness. Path *b* (the effect of loneliness on social adaptation) is not represented here, but was positive and significant (*p* < 0.001) in all models.*

In all models, lower levels of loneliness predicted greater social adaptation to university (i.e., path b: all βs < –0.39, all *p*s < 0.001). Examination of paths *a* and *c* in each model (i.e., daydreaming predicting loneliness and social adaptation) largely reflects previous analyses that constructive daydreaming predicts less loneliness but not social adaptation. Note that the effects for how lonely daydreams make participants feel and relationship quality are now marginal and non-significant, respectively. Of critical interest were paths *ab* (i.e., the indirect effects of daydreaming on social adaptation via loneliness), which were significant for daydreams that made participants feel more connected (β = 0.12, *SE* = 0.06, *p* = 0.047, 95% CI[0.02, 0.22]) and positive (β = 0.14, *SE* = 0.06, *p* = 0.033, 95% CI[0.03, 0.24]), marginally significant for daydreams that made participants feel less lonely (β = –0.13, *SE* = 0.07, *p* = 0.077, 95% CI[–0.24, –0.01]), and were more positive in content (β = 0.16, *SE* = 0.07, *p* = 0.013, 95% CI[0.06, 0.27]), and less fanciful (β = –0.19, *SE* = 0.09, *p* = 0.024, 95% CI[–0.35, –0.05]). This suggests that although social daydreams did not exert direct effects on social adaptation, they had an indirect effect on social adaptation via their effect on loneliness.

## Discussion

The evidence from this experience-sampling study supports the idea that the characteristics of social daydreaming during an important life context (the transition to university) promote adjustment to social challenges. First, consistent with the notion of an adaptive, but delayed, response to environmental challenges in which daydreams become more constructive in nature over time, the characteristics of daydreams became more constructive in the later, rather than earlier, weeks of the study. In the early weeks of the transition, no reliable patterns of change were observed in participants’ social daydreams but negative affect from the environment reliably increased during this time, indicating that the initial transition to university was a difficult period associated with negative feelings. In contrast, later study weeks were characterized by increasingly constructive social daydreaming over time; specifically, daydreams made participants feel more connected, less lonely, were less fanciful in nature and involved higher quality relationships. At the same time, participants also felt more connected with others, more positive, and less negative in general.

Second, participants who reported being less socially adapted to university showed faster changes in how connected their daydreams made them feel than others; that is, they showed less evidence of emotional inertia in response their social daydreaming. High emotional inertia is considered an index of maladjustment (e.g., Kuppens et al., 2010) suggesting that the lack of inertia for connectedness observed in participants who were less adapted to university was indicative of a functional affective response. Low emotional inertia is likely to reflect the adaptive nature of emotions, which enable individuals to flexibly respond to environmental challenges. Evidence of low emotional inertia for a positive social emotion (connectedness), as a result of cognition (social daydreaming) in a dynamic context (adjustment to university) contributes to the growing literature on the dynamics of emotion and adjustment (e.g., Kuppens et al., 2012). Our results indicate that high inertia is not necessarily a pattern of emotion dynamics for those who are currently socially maladjusted. Rather, current social maladjustment may be characterized by low inertia when individuals are in the process of adjusting to social challenges, which is likely to be functional (c.f., Koval and Kuppens, 2012).

Third, positive social daydreaming characteristics predicted less loneliness. Specifically, participants were less lonely if their social daydreams made them feel more connected, less lonely, and more positive and their content was less fanciful, more positively valenced, and involved higher quality relationships. These findings are consistent with a growing body of research showing that certain kinds of social daydreaming may buffer against or counteract loneliness (Mar et al., 2012; Poerio et al., 2013, 2015a). However, the present study extends these findings by demonstrating that social daydreaming can have a socio-emotional benefit over time during a naturally occurring period of social challenge.

Fourth, social daydreaming had an indirect effect on social adaptation to university via their influence on loneliness. Although we expected social daydreaming to directly predict social adjustment, this was not supported. However, supplementary mediation analyses showed indirect effects of daydreaming on social adaptation via loneliness for daydreams that made participants feel more connected, more positive, and less lonely and that were more positively valenced and less fanciful in content. These results suggest that social daydreaming may be especially linked to individuals’ socio-emotional well-being (e.g., loneliness) which then impacts on cognitive evaluations of their social situation. It is also possible that social daydreaming may have a longer-term effect on cognitive well-being (e.g., life satisfaction) which was not captured in the current month-long study.

### Mechanisms Linking Social Daydreaming to Adjustment

How does social daydreaming promote socio-emotional adjustment? Our findings point to the value of the regulation of social emotions (in particular feelings of social connection) for the process of adjustment. Over time, participants showed increases in feelings of interpersonal connection as a result of their social daydreams. Such an increase may be adaptive because it reflects a process whereby feelings of social connection contribute to more positive social interactions and the building of personal resources. Just as negative cognitions before and after social interactions (anticipatory and post-event processing, Clark and Wells, 1995) contribute to negative social interactions and the maintenance of social anxiety (e.g., Vassilopoulos, 2005; Taylor and Alden, 2011) we propose that an equivalent positive influence might be true for positive cognitions.

People who feel interpersonally connected after daydreaming may behave more positively toward others and have that positivity reciprocated in social interactions (Miller and Turnbull, 1986). Positive social interactions may lead to further feelings of social connection (Reis et al., 2000) and greater social resources (e.g., social support, interpersonal trust, and intimacy, Laurenceau et al., 1998; Burns et al., 2008; Kok and Fredrickson, 2010). Over time, the interplay between social interactions, social daydreaming and social emotions may contribute to greater socio-emotional functioning and greater socio-emotional well-being (e.g., less loneliness, flourishing).

In addition to emotional mechanisms, cognitive problem-solving processes might also explain, why social daydreaming promotes adjustment. Cognition can facilitate adjustment because it allows individuals to process important events, make sense of them and derive meaning (e.g., Taylor, 1983; Park, 2010). This involves problem-focused coping attempts that aid self-regulation and adjustment through the formation of concrete plans for action (Taylor et al., 1998). In particular, imagining past and possible future social interactions during social daydreams may facilitate learning, goal progress, problem-solving, and effective planning in the interpersonal domain (c.f., Baumeister and Masicampo, 2010).

Research on mental simulation and goals consistently shows that imagining the process, rather than the outcome, of goal achievement is associated with the successful pursuit of personal goals (Freund and Hennecke, 2015). That participants’ social daydreams became less fanciful over time suggests that daydreams eventually become more concrete and based on actual or probable social interactions and situations following a transition. This shift could be indicative of a more process-orientated approach to social problem-solving or planning which, in turn, may have facilitated later interpersonal behavior and reduced loneliness.

This view is consistent with evidence that daydreaming in general is associated with autobiographical planning (Baird et al., 2011) and social problem-solving (Ruby et al., 2013). However, whether or not social daydreams function in this manner for interpersonal goals is an open question. Research on the effect of mental simulation on goal achievement and coping has tended to focus on intrapersonal goals such as academic achievement or task performance (e.g., Pham and Taylor, 1999; Oettingen et al., 2000; Vasquez and Buehler, 2007) rather than on interpersonal goals such as the formation and maintenance of positive social relationships.

### Limitations and Future Directions

A skeptical reader might question whether our results simply reflect the process of adjustment rather than contributing to it. Of course, daydreams will, in part, reflect one’s current state of adjustment and the correlational nature of our investigation cannot unequivocally rule out reverse causation or third variable explanations. However, our analyses examining how social daydreams predicted later loneliness (and social adaptation via loneliness) controlled for these variables during the preceding 2 weeks thereby attenuating this concern. Whether or not social daydreams causally contributed to socio-emotional adjustment depends on whether imagination has a causal impact on later behavior and emotion. Various lines of research (reviewed in Baumeister et al., 2011) strongly suggest that conscious thought causes behavior albeit not immediately or directly, but the process by which social daydreams causally affect social behavior is a key question for future research. If this causal relationship is not supported, then we still have an epiphenomenon that is a potentially useful indicator of adjustment.

The present study only examined social daydreaming within one context of adjustment. We chose the university transition because it represents a stressful life event that is particularly associated with socio-emotional challenges. Whether similar findings can be observed during different life transitions should be addressed in future research. However, we expect that the theoretical rationale for why social daydreaming predicts adjustment would apply to various types of transitions where social goals and needs are pertinent (e.g., bereavement, marriage, divorce, parenthood). We also only examined social daydreaming at the start of a transition and could not therefore consider the anticipatory effects of daydreaming. However, anticipatory coping may occur before a stressful event, particularly when the event is expected, as in the case of the university transition (Aspinwall and Taylor, 1997). We would therefore expect social daydreaming in the weeks preceding a transition to be associated with adjustment as a form of pro-active coping (e.g., mental preparation for upcoming social interactions, thoughts about leaving established social networks, and expectations for the transition). Productive and unproductive social daydreaming in relation to an anticipated stressful event may be associated with later adjustment or maladjustment, respectively (e.g., Feldman and Hayes, 2005). In addition, it would have been informative to examine how dispositional characteristics of participants such as their pre-existing levels of depression or anxiety affected patterns of social daydreaming and moderated associations between daydreaming characteristics and loneliness. Given previous research linking dispositional affect to daydreaming (Marchetti et al., 2015) and the transition to university (Bewick et al., 2010), examining patterns of social daydreaming in potentially vulnerable participants could be particularly useful for examining for whom and when social daydreaming is associated with positive and negative outcomes in the context of life transitions and adjustment.

In addition to not examining social daydreaming prior to the university transition, we also only examined the early stages of that transition. This was based on the assumption that the first months would be especially likely to capture both the reaction and initial adaptive response to the transition. However, the potential effects of social daydreaming on adjustment may show different effects when examined over a longer time period. For example, we observed linear change in daydreaming characteristics over the latter weeks of the study, but longer sampling periods might reveal non-linear forms of change such as positive relationships that become weaker over time. It would be informative to examine the dynamics of social daydreaming over extended periods of time to adequately characterize the nature and form of change and how it relates to adjustment. Knowing the trajectory of social daydreaming in relation to adjustment could help to inform the timing of possible interventions directed at addressing social daydreaming to enhance socio-emotional well-being.

Despite these limitations, the present study offers a number of significant contributions to research and theory on daydreaming and adjustment. This is the first study to examine daydreaming repeatedly over time in the context of naturally occurring adjustment, showing that it is associated with an adaptive response. It is also the first study to examine the emotional dynamics of the outcomes of cognition by linking daydreaming with emotional inertia, which may be important for understanding the conditions under which cognition and emotion interact to predict adjustment. Finally, this study is the first systematic investigation of how the *social* content of thought is associated with adjustment. It is notable that previous research and theory on daydreaming and repetitive thinking have focused primarily on self-focused thoughts. Our research highlights the importance of exploring cognition that is focused on *others*, rather than just on the self, which is especially important given the amount of time spent thinking about others. Overall, we believe that this study represents the first step in developing an account of how imagining others facilitates adjustment and ultimately contributes to burgeoning evidence that, under the right conditions, letting the mind wander is functional.

## Author Contributions

GP conceived of and designed, the study in consultation with PT, L-ME, and EM. GP conducted the study and analyzed the data with assistance and contributions from PT. GP drafted the manuscript with contributions from PT, L-ME, and EM. All authors read and approved the final manuscript.

## Conflict of Interest Statement

The authors declare that the research was conducted in the absence of any commercial or financial relationships that could be construed as a potential conflict of interest.

The reviewer, Eve-Marie Blouin-Hudon, and handling Editor declared their shared affiliation, and the handling Editor states that the process nevertheless met the standards of a fair and objective review.
