# Corrigendum: Social Daydreaming and Adjustment: An Experience-Sampling Study of Socio-Emotional Adaptation During a Life Transition

**DOI:** 10.3389/fpsyg.2016.00174

**Published:** 2016-02-12

**Authors:** Giulia L. Poerio, Peter Totterdell, Lisa-Marie Emerson, Eleanor Miles

**Affiliations:** ^1^Department of Psychology, University of YorkYork, UK; ^2^Department of Psychology, University of SheffieldSheffield, UK; ^3^School of Psychology, University of SussexBrighton, UK

**Keywords:** daydreaming, mind wandering, socio-emotional adaptation, social cognition, social emotion, emotional inertia, loneliness, experience-sampling

The second and third authors affiliation was incorrectly stated as both the University of York and the University of Sheffield, which requires amendment (i.e., these authors are affiliated to the University of Sheffield only and not the University of York). This correction does not in any way affect the scientific validity of the results.

## Author contributions

GP conceived of, and designed, the study in consultation with PT, LE, and EM. GP conducted the study and analyzed the data with assistance and contributions from PT. GP drafted the manuscript with contributions from PT, LE, and EM. All authors read and approved the final manuscript.

## Funding

This research was supported by the Economic and Social Research Council [grant number ES-J500215-1] awarded to GP whilst at the University of Sheffield.

### Conflict of interest statement

The authors declare that the research was conducted in the absence of any commercial or financial relationships that could be construed as a potential conflict of interest.

